# Low-Temperature Co-Fermentation of *Lactobacillus bulgaricus 134* and *Saccharomyces cerevisiae*: Effects on Polyphenols Composition, Flavor Compounds and Antioxidant Activity of Black Rice Slurry

**DOI:** 10.3390/foods15112036

**Published:** 2026-06-05

**Authors:** Zuoting Xu, Chunlin Nie, Zhong Chen, Bingjie Liu

**Affiliations:** 1 School of Food Science and Engineering, South China University of Technology, Guangzhou 510641, China; fetunmux@mail.scut.edu.cn (Z.X.); ncl1754229326@163.com (C.N.); lbjie001@163.com (B.L.); 2 Dongguan Shilong Jinwei Beverage & Food Co., Ltd., Dongguan 523320, China

**Keywords:** low temperature, co-fermentation, polyphenol, flavor compounds, antioxidant capacity

## Abstract

Black rice is abundant in polyphenolic antioxidants, but conventional thermal processing degrades these heat-sensitive compounds, limiting their bioactivity. Although single-strain fermentation can improve the extraction of bioactive components, it remains challenging to simultaneously balance the flavor and bioactivity of fermented black rice products. Low-temperature co-fermentation with yeast and *lactobacillus* has emerged as a promising strategy to enhance both the flavor profile and functional quality of fermented foods. Therefore, this study investigates the effects of low-temperature co-fermentation with *Saccharomyces cerevisiae* and *Lactobacillus bulgaricus 134* on the quality of black rice slurry. The efficacy was systematically evaluated by monitoring fermentation kinetics, conducting polyphenol and anthocyanin metabolomics analysis, performing flavoromics analysis, and combining in vitro ABTS radical scavenging assays with a Caco-2 cell-based oxidative stress model. The results showed that this process activated β-glucosidase within the first 24 h of fermentation. By activating terpenoid and phenolic metabolic pathways, it maximized the accumulation of anthocyanins and short-chain esters during 30–36 h, which conferred the product with prominent fruity and sweet notes. Fermented black rice slurry (FBRS) exhibited potent ABTS radical scavenging activity. In the Caco-2 oxidative stress model, FBRS pretreatment restored cellular viability, upregulated the activity of endogenous antioxidant enzymes, and reduced MDA content. This study provides a theoretical foundation for developing high-nutritional, flavor-enhanced fermented black rice products.

## 1. Introduction

Black rice, a characteristic coarse cereal native to China, has been utilized in traditional food and wine production since the Shang and Zhou Dynasties. Compared with conventional colorless or glutinous rice, black rice contains significantly higher levels of anthocyanins, flavonoids, flavanones, and flavanols [1]. Consequently, black rice and its derived products have been proven to exert multiple health-promoting effects, including antioxidant, hypoglycemic, cholesterol-lowering, and anti-hyperuricemic activities [2,3,4,5]. The underlying mechanism is attributed to the redox activity of phenolic hydroxyl groups, especially para- and ortho-position hydroxyls, while the acidic structure of polyphenols further endows them with antioxidant capacity and favorable sensory properties [6].

In conventional food processing, black rice is mainly used for steaming and cake-making, as well as brewing. However, traditional thermal processing causes a rapid loss of heat-sensitive polyphenols and anthocyanins in black rice [7], which has driven the exploration of novel processing technologies to preserve and enhance its nutritional value. Fermentation has emerged as a promising strategy to improve the bioactivity of black rice. Existing studies have confirmed that fermentation can not only maintain or increase the content of beneficial phytochemicals, but also reduce unpleasant volatile substances [8], while producing high concentrations of aromatic alcohols, organic acids and esters to form a distinctive flavor profile [9]. Currently, *Lactobacillus plantarum* and *Saccharomyces cerevisiae* are the primary strains used for black rice fermentation. Yet single-strain fermentation cannot resolve the core trade-off between nutrition and flavor: *S. cerevisiae* fermentation improves anthocyanin color stability [10] and increases total phenolic content, but it reduces total flavonoid and anthocyanin levels [11]. Notably, after *S. cerevisiae* fermentation, anthocyanins in black rice can be converted into glycosides and free forms, with antioxidant capacity and β-glucosidase activity both reaching peak levels [12]. While *L. plantarum* fermentation enhances antioxidant capacity and protects anthocyanins [13,14], it often fails to form a balanced flavor profile.

To address this trade-off, multi-strain co-fermentation has been proposed to integrate individual strain advantages, enabling a simultaneous enhancement of nutrition and flavor [15]. Furthermore, low-temperature fermentation, which has been widely applied in wine production to improve flavor and protect heat-sensitive compounds [16,17], has been proven to further enhance the stability of anthocyanins. This technology can also regulate the bioconversion of phenolic compounds and stimulate *S. cerevisiae* to produce terpenoids, esters and other aromatic compounds [18,19,20,21,22]. However, despite its proven potential, low-temperature co-fermentation remains largely underexplored in black rice processing, with no systematic studies investigating its effects on the metabolic profile and bioactivity of fermented black rice products.

Therefore, this study aims to address the limitations of conventional black rice fermentation, and clarify the regulatory effect of low-temperature co-fermentation with *S. cerevisiae* and *L. bulgaricus 134* on the nutritional and flavor quality of black rice slurry. To achieve this aim, we conducted co-fermentation at 20 °C to produce fermented black rice slurry (FBRS), and performed a systematic evaluation: first, we analyzed the basic fermentation characteristics including pH, reducing sugar content, viable cell count and β-glucosidase activity; then, we performed flavoromics and metabolomics analyses to characterize the dynamic changes in polyphenols and anthocyanins; finally, we evaluated the antioxidant activity and cellular repair capacity via in vitro ABTS radical scavenging assay and a Caco-2 cell-based oxidative stress model.

## 2. Materials and Methods

### 2.1. Plant Materials and Cell Culture

Wuchang black rice (containing 4.2% dietary fiber, 73.9% carbohydrates, 2.3% lipids and 7.6% protein) was purchased from Chaihuo Courtyard Catering Co., Ltd. (Harbin, China). High-sugar-tolerant yeast (*S*. *cerevisiae*, 1 × 10^10^ CFU/g) was purchased from Angel Yeast Co., Ltd. (Wuhan, China). *L*. *bulgaricus 134* (1.5 × 10^10^ CFU/g) and Caco-2 cells (ATCC Cell Line, accession numbers: ATCC HTB-37) were obtained from the College of Food Science and Engineering, South China University of Technology. The strain is supplied in powder form and requires only activation at the appropriate fermentation temperature; no other pretreatment is necessary. The live cell counts of the *S*. *cerevisiae* and *L*. *bulgaricus 134* were determined simultaneously. The procedure is briefly described as follows: The bacterial powder was diluted to the appropriate dilution factor using MRS liquid medium, and the resulting suspension was then streaked onto MRS agar medium. After 24 h, the total number of colonies was counted, with each colony representing a single diluted cell.

Complete cell culture medium was prepared by supplementing DMEM (Dulbecco’s Modified Eagle Medium) with 10% fetal bovine serum, 100 U/mL penicillin, and 100 μg/mL streptomycin. Cryopreserved cells were rapidly thawed in a 37 °C water bath with gentle swirling. The entire thawed suspension was transferred to a centrifuge tube containing 10 mL of complete medium, mixed gently, and centrifuged at 1000× *g* for 3 min. After discarding the supernatant, the cell pellet was resuspended in 3–4 mL of fresh complete medium and seeded into a 6 cm culture dish. Cells were incubated at 37 °C in a 5% CO_2_ atmosphere.

### 2.2. Chemicals and Other Materials

Mesotherm α-amylase (A890337, 2000 U/g), chromatography-grade potassium bromide, ρ-nitrophenyl-β-D-glucopyranoside (ρNPG), and LC/MS reference standards (vitexin, quercetin, rutin) were purchased from Sigma-Aldrich Trading Co., Ltd. (Shanghai, China). ABTS stock solution, fetal bovine serum, penicillin-streptomycin dual resistance, DMEM, pancreatic enzyme and other related reagents were purchased from Gibco Life Sciences Co., Ltd. (Shanghai, China). SOD detection kit was purchased from Beijing Solarbio Science & Technology Co., Ltd. (Beijing, China). MDA (R21869-100T) and CAT (R41811-100T) detection kits were purchased from Yuanye Bio-Technology Co., Ltd. (Shanghai, China). All other chemical reagents used were of analytical grade.

### 2.3. Preparation of Sample Extracts

The rice is ground into powder using the FW-135 Herbal Medicine Crusher (Tianjin City Taisite Instrument Co., Ltd., Tianjin, China). Then, it is sieved through a 40-mesh screen, dry-sterilized at 160 °C for two hours using DHG-9240A Electric Forced-Air Drying Oven (Shanghai Qixin Scientific Instruments Co., Ltd., Shanghai, China), cooled, and stored in a refrigerator at 4 °C.

Black rice flour was mixed with sterilized distilled water at a mass ratio of 1:4. Based on preliminary experimental results, enzymatic hydrolysis was performed using 5% (*w*/*w*) mesotherm α-amylase (2000 U/g) at 55 °C for 1 h. The enzyme was inactivated by boiling in a water bath for 5 min, then the mixture was cooled to room temperature and adjusted to the preset fermentation temperatures (15, 20, 25 and 37 °C) according to the method of Melisa et al. [23]. *S. cerevisiae* and *L. bulgaricus 134* were inoculated at a viable cell count ratio of 2:3 (optimized via preliminary experiments), with a final inoculation density of 10^7^ CFU/mL (inoculum amount determined by weighing the bacterial powder based on pre-established viable count). Fermentation was carried out at the above four temperatures in an RQH-250Y Artificial Climate Chamber (Shanghai Jinghong Experimental Equipment Co., Ltd., Shanghai, China). Samples were collected at 0, 6, 12, 24, 30, 36 and 48 h, and the fermented black rice slurry (FBRS) was centrifuged at 9000 RCF and 4 °C for 10 min using an H2500R Refrigerated Centrifuge (Cence Laboratory Instrument Development Co., Ltd., Changsha, China) to separate the supernatant. After syringe filtration (0.22 μm pore size), the extracted samples were stored at 4 °C for subsequent analysis. The remaining centrifuged supernatant was pre-frozen at −20 °C for 24 h and then freeze-dried for 40 h; the obtained freeze-dried powder was used for polyphenol content determination.

### 2.4. Physicochemical Analysis

After the fermentation was completed, the pH of the sample was immediately measured using a Leici pH S-25 pH meter (Shanghai Precision Scientific Instrument Co., Ltd., Shanghai, China) with a two-point calibration procedure. Total titratable acidity (TTA) was determined according to National Standard of China GB 12456-2021 [24], via titration with 0.1 mol/L NaOH using phenolphthalein as the indicator. Reducing sugar content was measured via the 3,5-dinitrosalicylic acid (DNS) method in accordance with GB 5009.7-2016 [25]. A standard curve of absorbance versus reducing sugar concentration was established with DNS as the chromogenic reagent, and sample absorbance was detected using a UV-1800 UV-Vis spectrophotometer (Shimadzu Instruments Manufacturing Co., Ltd., Suzhou, China) for content calculation.

### 2.5. Determination of Polyphenol Content

Polyphenol determination employs ultraviolet spectrophotometry. A total of 5 g of freeze-dried FBRS powder was weighed and soluble in 10 mL of an 80% ethanol solution. The solution was sonicated for 60 min with shaking occasionally to maintain solid dispersion. After centrifuge at 7655 RCF at 4 °C for 5 min, the supernatant was collected, the residue was rinsed twice with alcohol, diluted to 25 mL, and stored at −20 °C protected from light. To perform alkaline hydrolysis, 20 mL of 4 mol/L NaOH was added to the centrifuge residue, by shaking at 37 °C and 130 rpm for 2 h. After hydrolysis, the pH of the hydrolysate was adjusted to 2.0 with hydrochloric acid. The hydrolysate was extracted three times with 60 mL of ethyl acetate. The organic layers were combined and evaporated to dryness at 40 °C using a rotary evaporator. The resulting extract was redissolved in 70% ethanol and diluted to a final volume of 10 mL to obtain the bound phenol extract, which was then stored sealed and protected from light at −20 °C. For analysis, 1 mL of the extract was transferred into a 25 mL graduated test tube, followed by the addition of 1 mL of 1 M Phlorizin and 6 mL of deionized water. The mixture was shaken thoroughly and allowed to stand for 5 min. Subsequently, 4 mL of a 10% sodium carbonate solution was added, and the reaction was left to stand at room temperature for 60 min. The mixture was then diluted to the mark with deionized water and shaken well. The absorbance of the resulting solution was measured at 760 nm.

### 2.6. Determination of Anthocyanin Content

Mix the centrifuged sample produced in 2.3 with buffers at pH 1.0 and pH 4.5 in a 1:9 ratio. Measure the absorbance at 510 nm and 700 nm, respectively, and calculate using the following formula:

$$\mathrm{Anthocyanin}\;(\mathrm{cyanidin}-3-\mathrm{glucoside}\;\mathrm{equivalents},\;\mathrm{mg}/L)=\frac{A\times MW\times DF\times 10^{3}}{\varepsilon \times 1}$$

A = (A_510nm_ − A_700nm_) pH 1.0 − (A_510nm_ − A_700nm_) pH 4.5;

MW = molecular weight of cyanidin-3-glucoside (MW = 449.2 g/mol);

DF = dilution factor;

1 = light path length of 1 cm;

ε = 26,900, the molar extinction coefficient of cyaniding-3-glucoside;

10^3^ = factor converted from g into mg.

Based on the results of determining the fundamental fermentation characteristics, subsequent experiments selected 20 °C as the optimal condition for fermentation.

### 2.7. Determination of Changes in Live Bacteria Count in Samples During Fermentation

The changes in the number of viable bacteria during fermentation are determined using the plate count method. After appropriately diluting the sample, 0.1 mL of the diluted solution is spread onto an agar plate. Following incubation, each individual cell grows and multiplies to form a colony visible to the naked eye; that is, a single colony should represent a single cell in the original sample. The number of colonies is counted, and the bacterial count in the sample can be calculated based on the dilution factor and the inoculum volume. By adding ampicillin and natamycin to MRS agar, *S. cerevisiae* and *L. bulgaricus 134* can be counted, respectively.

### 2.8. Determination of β-Glucosidase Activity

The activity of β-glucosidase was determined according to the method of Chaiyasut et al. with some modifications [12]; β-glucosidase activity was assessed by measuring the rate of hydrolysis of ρNPG. In brief, 50 μL of sample, 100 μL of 3.3 mM ρNPG and 200 μL of 0.5 M Na_2_CO_3_ were mixed. The same mixture without sample was used as a blank. It was then incubated at 40 °C for 25 min and measured at 405 nm using a microplate reader. β-Glucosidase activity is expressed as a percentage of relative activity, calculated according to the following formula:

$$\mathrm{Clearance}\;\mathrm{rate}=\frac{A_{1}-A_{n}}{A_{1}}$$

*A*_1_ = absorbance value of the blank control group;

*A_n_* = absorbance value of the sample group.

### 2.9. Quantitative Analysis of Polyphenol and Anthocyanin Content

The data acquisition instrument system primarily consists of ultra-high performance liquid chromatography (UPLC Vanquish, Thermo Inc, Atlanta, GA, USA) and high-resolution mass spectrometry (Q Exactive, Thermo Inc, Atlanta, GA, USA). The chromatographic column is a Waters HSS T3 (50 × 2.1 mm, 1.8 μm).

#### 2.9.1. Analysis of Polyphenol Content Using LC/MS

UPLC was conducted based on a previously published method adopting the following conditions: mobile phase A, 0.1% formic acid ultra-pure water solution; mobile phase B, 0.1% formic acid acetonitrile solution; mobile phase gradient conditions: (1) 0–2 min A/B (90:10, *v*/*v*), (2) 2–6 min A/B (90:10, *v*/*v*), (3) 6–9 min A/B (40:60, *v*/*v*), (4) 9–9.1 min A/B (40:60, *v*/*v*), (5) 9.1–12 min A/B (90:10, *v*/*v*), (6) 12 min A/B (90:10, *v*/*v*). Flow rate: 0.3 mL/min; column temperature: 40 °C; injection volume: 2 μg. The entire analysis process was conducted with the sample placed in an automatic sampler at 4 °C. To improve testing efficiency, MS conditions were as follows: negative ion detection mode; sheath gas 40 arb, auxiliary gas 10 arb, ion spray voltage −2.8 kV, temperature 350 °C, and ion transfer tube temperature 320 °C; ion scanning range, 100–900 (*m*/*z*) [26].

#### 2.9.2. Analysis of Anthocyanin Content Using LC/MS

Since anthocyanins are key components of black rice’s nutritional profile, a separate analysis of anthocyanin flavoromics was conducted in addition to the polyphenol metabolomics study to enhance the accuracy of our results. UPLC was conducted based on a previously published method adopting the following conditions [27]: mobile phase A, 0.1% formic acid ultra-pure water solution; mobile phase B, 0.1% formic acid acetonitrile solution; mobile phase gradient conditions: (1) 0–1 min A/B (95:5, *v*/*v*), (2) 1–6 min A/B (95:5, *v*/*v*), (3) 6–7 min A/B (70:30, *v*/*v*), (4) 7–8 min A/B (5:95, *v*/*v*), (5) 8–8.1 min A/B (5:95, *v*/*v*), (6) 8.1–10 min A/B (95:5, *v*/*v*), (7) 10 min A/B (95:5, *v*/*v*). Flow rate: 0.3 mL/min; column temperature: 40 °C; injection volume: 2 μg. The entire analysis process was conducted with the sample placed in an automatic sampler at 4 °C. To improve testing efficiency, MS conditions were as follows: positive ion detection mode; sheath gas 40 arb, auxiliary gas 10 arb, ion spray voltage 3 kV, temperature 350 °C, and ion transfer tube temperature 320 °C; ion scanning range, 200–700 (*m*/*z*). The sample was kept at 4 °C in an automatic sampler throughout the analysis process [28].

#### 2.9.3. Analysis of Flavor Components Using GC/MS

The Gas Chromatography–Mass Spectrometry (GC/MS) method following the methodology of Wu et al. [7,29,30,31] was used; an aliquot of the fermented black rice sample was placed in a 20 mL headspace vial, maintained at 60 °C, and agitated for 5 min. A 120 µm DVB/CWR/PDMS fiber (Supelco, Bellefonte, PA, USA) was conditioned at 250 °C for 5 min prior to extraction. The fiber was then inserted into the headspace vial and exposed for 15 min at 60 °C to extract the volatile compounds. Subsequently, the fiber was desorbed in the GC inlet at 250 °C for 5 min. GC-MS analysis was performed using an Agilent 7000D system (Agilent Inc, Santa Clara, CA, USA) equipped with a DB-5MS capillary column (30 m × 0.25 mm × 0.25 µm; Agilent J&W Scientific, Folsom, CA, USA). Helium was used as the carrier gas at a constant flow rate of 1.2 mL/min. The inlet temperature was set at 250 °C, and the injection was performed in spitless mode with a solvent delay of 3.5 min. The oven temperature program was as follows: initial temperature held at 40 °C for 3.5 min, increased to 100 °C at 10 °C/min, then raised to 180 °C at 7 °C/min, and finally held at 180 °C for 5 min. The mass spectrometer was operated in electron impact (EI) mode at 70 eV. The ion source temperature was 230 °C, the quadrupole temperature was 150 °C, and the interface temperature was 280 °C. Data acquisition was performed in selected ion monitoring (SIM) mode, and qualitative and quantitative ion monitoring was conducted in accordance with the provisions of GB/T 15038-2006 [32]. The constituents were identified based on two criteria. First, a mass spectral similarity scores greater than 800 was required against the NIST 14 database. Second, the calculated retention index (RI) for each compound had to be within ±50 of the reference RI in the NIST library. After preprocessing, normalization, and quality control of the raw GC-MS data, unsupervised principal component analysis (PCA) was first performed to examine the overall metabolic profiles of the samples and the separation trends between groups. Hierarchical cluster analysis (HCA) was then used to validate the grouping results and analyze metabolite expression patterns; subsequently, a supervised Orthogonal Partial Least Squares Discriminant Analysis (OPLS-DA) model was constructed. After confirming the model’s reliability through cross-validation and bootstrap testing, differential metabolites were screened using VIP values and *t*-tests. Finally, the differential metabolites were mapped to the Kyoto Encyclopedia of Genes and Genomes (KEGG) database for metabolic pathway enrichment analysis to identify key metabolic pathways and regulatory mechanisms associated with the experimental intervention.

### 2.10. ABTS^+^ Scavenging Activity

The ABTS^+^ and DPPH·scavenging activity was determined with reference to China GB/T 39100-2020 [33] *Determination of antioxidant activity for polypeptides-DPPH and ABTS methods*, and the results were expressed as scavenging rate. The prepared and calibrated PBS buffer should then be added to the ABTS working solution to adjust its absorbance to 0.70 ± 0.02, thereby obtaining the ABTS experimental solution. A 96-well plate should be utilized, with 50 μL of sample solution and 100 μL of ABTS^+^ added to each well. The plate should then be mixed and incubated for 30 min. The absorbances at 734 nm should then be measured, with three replicate sets for each group. As for the DPPH method, accurately prepare 0.1 mM DPPH working solution in methanol, which should be freshly prepared and strictly stored in the dark. Dissolve 0.5 g of the lyophilized powder obtained in Section 2.3 in 10 mL of methanol to prepare the test solution. Add the DPPH solution first, followed by the sample solution to avoid local over-concentration. Set up blank control (DPPH + solvent) and sample background control (sample + solvent) to eliminate interference from solvent and sample color. Incubate the mixture strictly in the dark at room temperature for 30 min. Measure the absorbance at 517 nm using a UV-1800 UV-Vis spectrophotometer, with three replicates per group.

### 2.11. Cell Experiments

#### 2.11.1. Grouping and Dosing

Caco-2 cells were divided into three groups according to different fermentation temperatures. Each group included a blank control, a negative control, and treatment groups corresponding to different fermentation time points (0, 6, 12, 24, 30, 36, and 48 h). Cells in the logarithmic growth phase were seeded into culture plates. After overnight incubation, the culture medium containing various concentrations (1, 2, 5, 10 percent) of FBRS was applied to the treatment groups, while the blank control and negative control groups received an equal volume of fresh medium; the concentration of FBRS added was determined based on the results of preliminary experiments. After 24 h, with the exception of the blank control group, all other groups were treated with 500 μM H_2_O_2_. The blank control group was supplemented with an equivalent volume of culture medium. All cells were further incubated for 6 h prior to subsequent assays.

#### 2.11.2. Cell Viability Assay

Caco-2 cells were seeded into a 96-well plate at a density of 1.8 × 10^4^ cells per well in 100 μL of medium. After treatment according to the aforementioned method, cell morphology was observed and photographed using inverted fluorescence microscope MF52-N (Guangzhou Mshot Optics Technology Co., Ltd., Guangzhou, China). Subsequently, 20 μL of MTT solution (5 mg/mL) was added to each well, followed by incubation for 4 h. The supernatant was then carefully aspirated, and 150 μL of DMSO was added to each well to dissolve the formazan crystals. The plate was shaken gently for 10 min, and the absorbance was measured at 490 nm using a microplate reader (SuPerMax 2800MF Multifunctional Microplate Reader, Shanpu Technology Co., Ltd., Beijing, China).

#### 2.11.3. Determination of Malondialdehyde, Superoxide Dismutase and Catalase

Cells in the logarithmic growth phase were harvested and seeded into 24-well plates at a density of 1.5 × 10^4^ cells per well. Following treatment according to the aforementioned method, the cell culture medium was collected and centrifuged for 5 min. Subsequently, 200 μL of the supernatant was aliquoted, and the levels of Malondialdehyde (MDA), Superoxide Dismutase (SOD), and Catalase (CAT) were determined using their respective assay kits.

### 2.12. Statistical Analyses

All experiments were performed in triplicate, and results were reported at a 95% confidence interval. Statistical analysis was conducted using SPSS (v. 23.0). One-way analysis of variance (ANOVA) followed by Fisher’s Least Significant Difference (LSD) post hoc test was employed to determine significant differences in the levels of anthocyanins, anthocyanidins, and phenolic acids across different fermentation time points.

## 3. Results and Discussion

### 3.1. Changes in pH, Total Titratable Acidity, Anthocyanin, and Polyphenol Content

**Figure 1:**
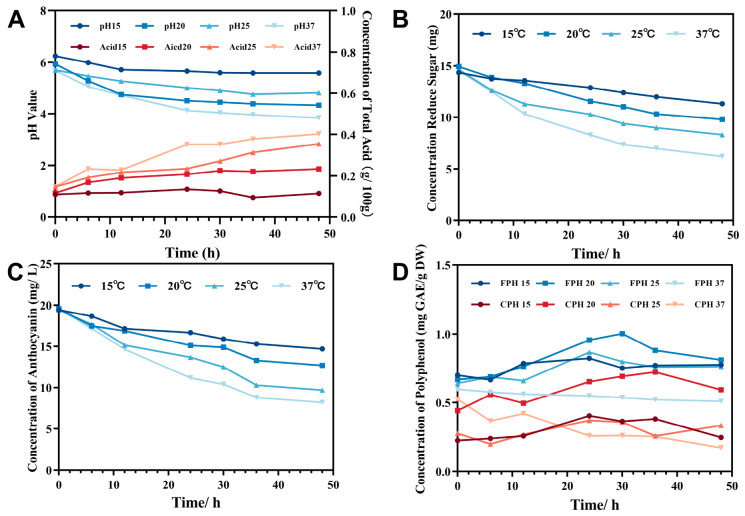
Fermentation kinetics and bioactive compound changes in FBRS at different temperatures. (**A**) pH value and total titratable acid (TTA) content; (**B**) reducing sugar concentration; (**C**) total anthocyanin concentration; (**D**) free polyphenol (FPH) and combined polyphenol (CPH) concentrations. A 48 h co-fermentation at 15, 20, 25, and 37 °C. FPH 15/20/25/37, free polyphenols in samples fermented at corresponding temperatures; CPH 15/20/25/37, combined polyphenols in samples fermented at corresponding temperatures. Data are presented as mean ± standard deviation (SD) of three independent biological replicates.

The fermentation characteristics of black rice slurry were systematically evaluated at 15, 20 and 25 °C, with 37 °C (the commonly recognized optimal growth temperature for the tested strains) set as the control. Among the tested temperatures, 20 °C was identified as the optimal fermentation condition. At 20 °C, the pH of the system decreased gradually (Figure 1A), indicating continuous acid production by the microorganisms. This condition effectively balanced the trade-off between excessively rapid acidification at higher temperatures (25 and 37 °C) and sluggish acid production at 15 °C. Reducing sugar consumption was the most moderate and stable at 20 °C (Figure 1B), while higher temperatures caused excessive sugar utilization for microbial proliferation. In addition, fermentation at 20 °C minimized thermal degradation of anthocyanins (Figure 1C), with anthocyanin content remaining relatively stable or only decreasing gradually during the mid-to-late fermentation stages. These findings are consistent with previous reports that fermentation with *S. cerevisiae* and *Levilactobacillus brevis* LUC 247 increases the contents of anthocyanins, total phenolics and total flavonoids in black rice slurry [34,35].

Given the low polyphenol content and organic acid production at 15 °C, black rice slurry fermented at this temperature exhibited lower nutritional value; thus, 20 °C was determined as the optimal condition for total polyphenol extraction (Figure 1D). Significant and sustained accumulation of free phenolics was observed at 20 °C, confirming that microbial enzyme systems were activated and reached peak activity under this condition. Based on the above results, all subsequent experiments were performed at 20 °C to investigate the correlation between various fermentation parameters and fermentation time.

### 3.2. Viable Cell Count

**Figure 2:**
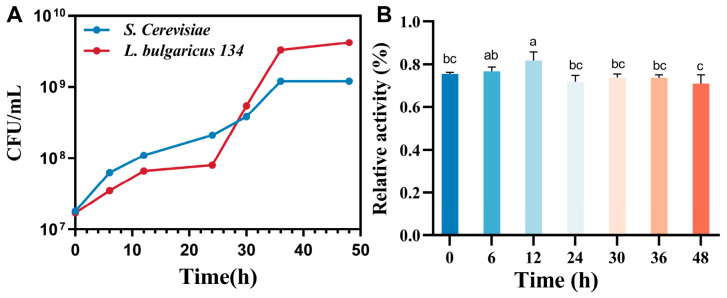
Viable cell counts and β-glucosidase activity during 20 °C co-fermentation. (**A**) Viable cell counts of *Saccharomyces cerevisiae* and *Lactobacillus bulgaricus* (CFU/mL); (**B**) β-glucosidase activity (U/mL). A 48 h co-fermentation at 20 °C. Different lowercase letters indicate significant differences among fermentation time points (*p* < 0.05). Data are mean ± SD of three independent biological replicates. The bar chart uses different colors solely to indicate differences in fermentation time, with no other meaning.

As shown in Figure 2A, the growth profiles of *S. cerevisiae* and *L. bulgaricus 134* during co-fermentation at 20 °C are presented. Throughout the fermentation cycle, the viable counts of both strains increased continuously and maintained at high levels. *S. cerevisiae* entered a rapid proliferation phase in the early fermentation stage (0–24 h), becoming the dominant strain in the system within this period, while *L. bulgaricus 134* grew at a slower rate. The viable count of *L. bulgaricus 134* increased steadily during the mid-to-late fermentation stages (24–48 h). This result indicates that *S. cerevisiae* had much stronger low-temperature adaptability than *L. bulgaricus 134*, which may be attributed to the differential expression of metabolic genes (PSD1, OPI3, ERG3, LCB3 and NTH1) of *S. cerevisiae* during low-temperature fermentation [36].

### 3.3. β-Glucosidase Activity

β-glucosidase activity results are shown in Figure 2B. β-glucosidase activity was maintained at a high level during the first 12 h of fermentation, followed by a significant decrease. The change in intracellular enzyme activity of *S. cerevisiae* is closely related to the accumulation of aglycone anthocyanins and the release of volatile terpenoid flavor compounds [12]. This indicates that β-glucosidase produced during co-fermentation is the key enzyme catalyzing the hydrolysis of bound flavor precursors and polyphenol–anthocyanin glycosides in black rice slurry, which directly contributes to the increase in anthocyanin and aromatic substance contents.

### 3.4. Anthocyanin Metabonomic

A targeted metabolomic approach was used to identify 10 anthocyanins and their derivatives, and the detected flavonoid compounds were categorized into four classes: flavones, flavonoid glycosides, hydroxyflavonoids, and O-methylated flavonoids. As shown in Table 1, total anthocyanin content peaked at 36 h of fermentation, accompanied by a marked increase in flavones and flavonoid glycosides, followed by a decline between 36 h and 48 h. This dynamic change was closely associated with the synergistic effects of *S. cerevisiae* and *L. bulgaricus*: *S. cerevisiae* promoted the release of anthocyanins and flavonoids via enzyme secretion, while *L. bulgaricus* regulated their stability. Analysis of representative anthocyanins showed that several increased after 36 h, pelargonidin declined, and petunidin remained stable, reflecting strain-specific regulatory differences in anthocyanin metabolism.

**Figure 3:**
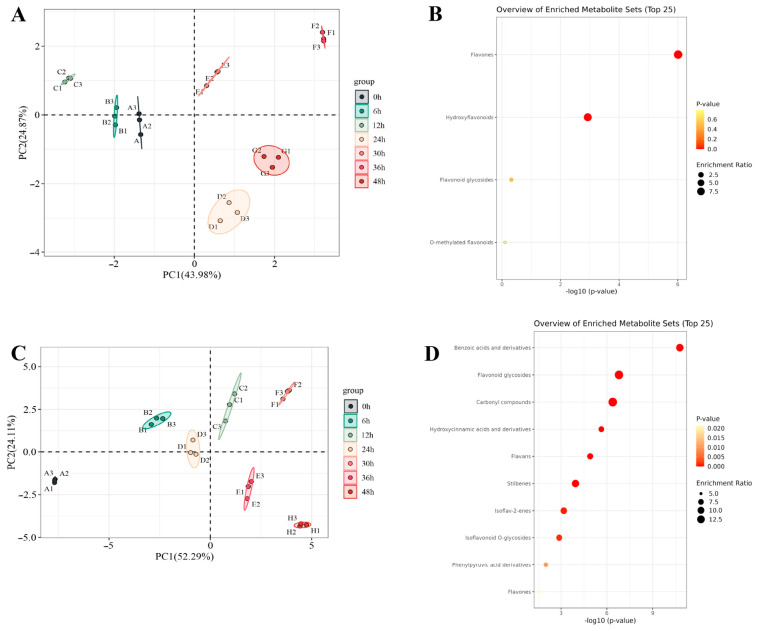
Metabolomic analysis of anthocyanins and polyphenols. (**A**) PCA score plot of anthocyanin metabolites; (**B**) metabolite set enrichment bubble chart of anthocyanin metabolites; (**C**) PCA score plot of polyphenol metabolites; (**D**) metabolite set enrichment bubble chart of polyphenol metabolites. Samples collected at 0, 6, 12, 18, 24, 30, 36, and 48 h (triplicate per time point). For PCA, first two principal components explain 52.29% and 24.11% of total variance (cumulative 76.40%). Bubble size, number of enriched metabolites; bubble color, enrichment significance (darker = lower *p*-value). Data from three independent biological replicates.

Principal component analysis (PCA) results (Figure 3A) reveal that the first two principal components accounted for 52.29% and 24.11% of the total variance (cumulative 76.40%), effectively representing overall anthocyanin metabolic variation. Triplicate samples at the same time point aggregated closely, while different fermentation stages separated completely, confirming that fermentation time drove significant dynamic changes jointly mediated by *S. cerevisiae*-induced metabolic remodeling and *L. bulgaricus*-dependent substrate conversion.

Metabolite set enrichment analysis (Figure 3B) identified benzoic acids and derivatives as the most significantly enriched category, followed by flavonoid glycosides, carbonyl compounds and hydroxycinnamic acids. This indicates fermentation mainly regulated benzoic acid derivatives, flavonoid glycosides and phenylpropane-related pathways, with *S. cerevisiae* primarily modulating flavonoid glycoside hydrolysis and *L. bulgaricus* driving the metabolism of benzoic and hydroxycinnamic acid derivatives.

Microbially derived β-glucosidase primarily secreted by *S. cerevisiae* is hypothesized to play a core role in anthocyanin modification, catalyzing the hydrolysis of anthocyanin glycosides to corresponding aglycones and potentially mediating further degradation of released anthocyanidin structures. Within the first 36 h, this hydrolysis driven by *S. cerevisiae* β-glucosidase drove marked accumulation of aglycone forms [12,13,37]. Notably, no cyanidin glycosides were detected, indicating increased cyanidin likely stemmed from enhanced extraction efficiency rather than in situ biotransformation [38]. Extending fermentation to 48 h reduced most bioactive compounds, likely driven by substrate exhaustion and *L. bulgaricus*-mediated utilization of aglycones as secondary carbon sources.

### 3.5. Polyphenol Metabonomic

A targeted metabolomics approach enabled the identification of 21 phenolic compounds and their derivatives, which were classified into six major categories: benzoic acids and derivatives, hydroxycinnamic acids and derivatives, flavans, flavonoid glycosides, phenylpyruvic acid derivatives, carbonyl compounds, flavones, isoflavone *O*-glycosides, isoflavon-2-enes, and stilbenes. Notably, the levels of flavans and flavonoid glycosides increased substantially following fermentation. However, an overall decline in free phenolic compounds was observed throughout the low-temperature fermentation process.

Changes in the levels of representative phenolic acids are summarized in Table 2. The concentrations of vanillic acid, epicatechin, and naringenin increased, whereas those of ferulic acid, 3,4-dihydroxybenzoic acid, and p-hydroxycinnamic acid decreased. Principal component analysis (PCA) was applied to characterize the overall metabolic changes in polyphenols during fermentation (Figure 3C). The first two principal components explained 43.98% and 24.87% of the total variance, respectively, with a cumulative interpretation rate of 68.85%, effectively reflecting the metabolic differences among samples at different fermentation time points. Triplicate samples from the same fermentation stage were tightly clustered, indicating reliable experimental repeatability, while samples from different fermentation durations were clearly separated, demonstrating that fermentation time induced a significant time-dependent shift in the polyphenol metabolic profile. Orthogonal Partial Least Squares Discriminant Analysis (OPLS-DA) was employed to discriminate between the metabolic profiles at the end of fermentation and those of the unfermented control. The model demonstrated strong discriminatory power, with Q^2^ = 0.4981 and R^2^Y = 0.9306. The principal differential metabolites identified, including 3,4-dihydroxybenzoic acid, protocatechualdehyde, p-hydroxycinnamic acid, salicylic acid, and ferulic acid, all exhibited decreased levels following fermentation.

Quantitative analysis of phenolic compounds showed that glycoside-bound phenolics decreased significantly during fermentation, accompanied by a transient accumulation of free aglycones including naringenin and epicatechin. This early-stage hydrolysis was driven by *S. cerevisiae*-derived β-glucosidase, matching our earlier fermentation kinetics results. The content of most free phenolic forms decreased continuously in the middle and late fermentation stages. This trend suggests that, in addition to limited isomerization and interconversion among free phenolic monomers, a proportion of free phenolics were re-converted to bound forms, or utilized as secondary carbon sources by *L. bulgaricus*, consistent with previously reported microbial phenolic metabolism patterns [19]. Metabolite set enrichment analysis (Figure 3D) further confirmed that flavones were the most significantly enriched metabolite category, followed by hydroxyflavonoids, flavonoid glycosides and O-methylated flavonoids, indicating that flavonoid subclasses were the core differentially regulated polyphenol metabolites during fermentation.

### 3.6. Flavor Component Content in Fermented Black Rice Slurry

#### 3.6.1. Sensory Flavor Characteristics of Differential Metabolites

**Figure 4:**
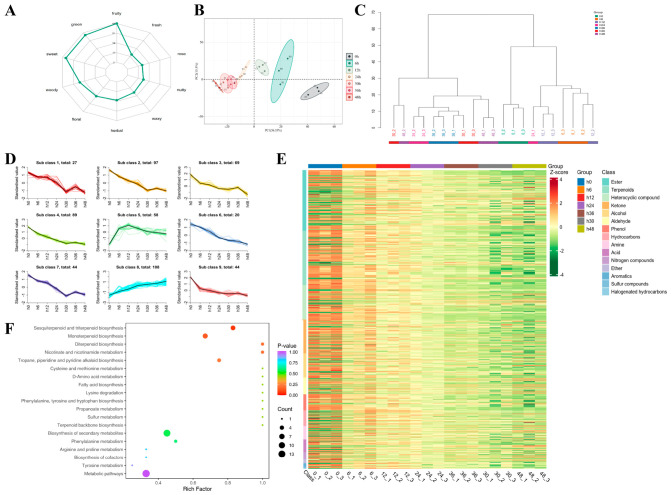
Flavor metabolomic profiling of FBRS. (**A**) Flavor profile radar chart; (**B**) PCA score plot of volatile flavor compounds; (**C**) hierarchical clustering tree of flavor compounds; (**D**) K-means clustering analysis of flavor compounds; (**E**) cluster heatmap of differentially expressed flavor metabolites; (**F**) pathway enrichment map of differential flavor metabolites. Samples collected at 0, 6, 12, 18, 24, 30, 36, and 48 h (triplicate per time point). Data from three independent biological replicates. The different colors in the K-means plot simply represent the differences between clusters and have no other meaning.

As shown in the sensory radar chart (Figure 4A), the fermented black rice slurry presented a complex flavor profile, dominated by herbal and fresh notes, accompanied by distinct sweet and rosy attributes, with waxy and grassy notes further enriching its flavor complexity. Concurrently, metabolic pathways related to aromatic amino acid metabolism, especially the biosynthesis and metabolism of phenylalanine, tyrosine and tryptophan, were significantly enriched. These amino acids are the core biosynthetic precursors of various phenolic and flavonoid antioxidants, including anthocyanins. The fermentation process formed a balanced and complex flavor profile, which was highly consistent with metabolomic data showing significant enrichment of terpenes and esters. Terpenes are widely recognized as the main contributors to herbal and floral aromas, while esters confer fruity and sweet notes [39,40]. Notably, the intensity of undesirable flavors such as pungency remained at a very low level, indicating that this fermentation process did not produce notable off-flavors.

#### 3.6.2. Principal Component Analysis (PCA)

Principal Component Analysis (PCA, Figure 4B) demonstrated that fermentation duration exerted a pronounced effect on the temporal evolution of the black rice slurry metabolome. Samples showed a clear time-dependent trajectory along Principal Component 1 (PC1), which explains 56.35% of the total variance. Notably, samples collected at 24, 30 and 36 h clustered closely, forming a distinct group indicative of a relatively stable metabolic phase. This indicates that the 24–36 h fermentation window was the critical period for metabolic transformation in black rice slurry, consistent with the peak accumulation of antioxidant and characteristic flavor compounds observed during this stage. In contrast, the 48 h sample deviated markedly from this cluster, indicating that prolonged fermentation may induce unfavorable alterations in the metabolic profile.

#### 3.6.3. Hierarchical Cluster Analysis (HCA)

Hierarchical cluster analysis (HCA) revealed that all samples were clearly divided into two major clusters based on fermentation time (Figure 4C). Early fermentation samples (0, 6, 12 h) clustered together, while late fermentation samples (24, 30, 36, 48 h) formed another major cluster. Notably, samples from 30 and 36 h exhibited the highest similarity, nearly overlapping completely in the cluster tree, indicating highly homogeneous metabolite compositions between them. This separation pattern corresponds to the dominant growth phases of the two bacterial strains observed in the viable cell count test results, and confirms that from an unsupervised analysis perspective, the fermentation time of 30–36 h represents the most stable and consistent metabolic state during black rice slurry fermentation.

#### 3.6.4. K-Means Analysis

K-means clustering analysis was employed to delineate the evolution patterns of volatile compounds over the fermentation time course. Nine distinct clusters were identified, as shown in Figure 4D. Cluster 5, corresponding to the early metabolic activity of *S. cerevisiae*, exhibited a rapid increase in content within the first 12 h of fermentation, and was mainly composed of esters of C1–C4 short-chain alcohols, with no fatty acids detected. Cluster 8, corresponding to the late metabolic activity of *L. bulgaricus*, showed an upward trend with decreasing fermentation temperature, and was dominated by organic acids, ethanol-derived esters, and terpenoids. The remaining clusters (1, 2, 3, 4, 6, 7 and 9) showed a downward trend over fermentation time, mainly including major terpenoids, partial esters and ketones. The metabolite distribution across the three change patterns indicates that low-temperature fermentation promoted the complementary metabolism of the two strains, increasing short-chain ester content and enhancing the aroma complexity of FBRS.

#### 3.6.5. Differential Metabolites Clustering Analysis

KEGG pathway enrichment analysis (Figure 4E) revealed that the fermentation process reshaped the metabolic network of the microbial fermentation system. As shown in the bubble plot, terpenoid biosynthesis pathways showed the most significant enrichment, with sesquiterpene/triterpene, monoterpene and diterpene biosynthesis pathways ranking at the top. This directly verified at the metabolic pathway level that fermentation significantly activated the key synthetic pathways of flavor and bioactive compounds in black rice slurry, which was consistent with the abundant accumulation of volatile terpenoids (key contributors to fruity and floral aromas) detected by GC-MS and sensory analysis. Meanwhile, aromatic amino acid metabolism pathways, including the biosynthesis and metabolism of phenylalanine, tyrosine and tryptophan, were also significantly enriched. These aromatic amino acids are the core synthetic precursors of multiple phenolic and flavonoid antioxidants (e.g., anthocyanins). Activation of these pathways provided a clear metabolic basis for the accumulation of polyphenols and anthocyanin aglycones during the mid-fermentation stage (24–36 h). In addition, enrichment of pathways including nicotinic acid metabolism and cofactor biosynthesis reflected the active primary metabolism of microorganisms during fermentation. In summary, differential metabolites were mainly enriched in the biosynthetic pathways of two major secondary metabolites, terpenoids and phenolics, which constituted the core metabolic mechanism underlying the improved flavor and nutritional quality of FBRS.

#### 3.6.6. Differential Metabolites Pathway Enrichment Analysis

KEGG pathway enrichment analysis of differential flavor metabolites (Figure 4F) revealed that fermentation systematically activated the biosynthetic networks of terpenoids and aromatic amino acids in *S. cerevisiae*, and promoted the biosynthesis and extracellular release of terpenoids [41]. Among these, sesquiterpene/triterpene and monoterpene biosynthesis pathways showed the most significant enrichment (*p* < 0.01). This verified at the metabolic pathway level that fermentation preferentially promoted the biosynthesis of terpenoid flavor compounds, which was fully consistent with the changes in terpenoid content detected by GC-MS.

### 3.7. ABTS Free Radical Scavenging Ability of Fermented Rice

**Figure 5:**
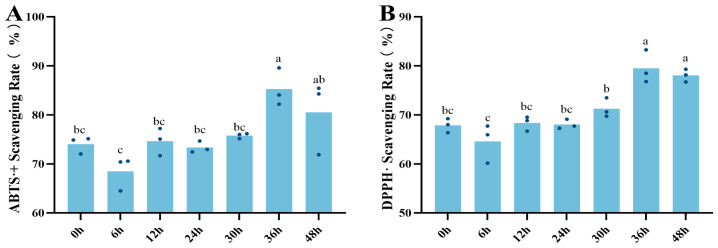
Antioxidant activity of FBRS during fermentation. (**A**) ABTS radical scavenging activity (%); (**B**) DPPH radical scavenging activity (%). A 48 h co-fermentation at 20 °C. Data are mean ± SD of three independent biological replicates. Different letters in the bar chart indicate significant differences between groups. Different dots in the figure represent different parallel experiments.

In vitro antioxidant activity of FBRS extracts was evaluated by ABTS^+^ and DPPH· radical scavenging assays (Figure 5A,B). Both assays showed highly consistent temporal patterns: scavenging activity decreased significantly at 6 h (*p* < 0.05), recovered gradually from 12 h to 30 h, peaked at 36 h, and slightly declined at 48 h.

DPPH· scavenging rates were consistently 6–12% lower than ABTS^+^ values across all time points, reflecting their mechanistic differences: ABTS^+^ measures total antioxidant capacity via electron transfer, detecting both water- and lipid-soluble antioxidants, while DPPH· primarily reflects hydrogen atom transfer ability of lipophilic polyhydroxylated compounds. At 36 h, both assays showed significantly higher activity than earlier time points (*p* < 0.05), with ABTS^+^ and DPPH· scavenging rates reaching 85.29 ± 3.82% and 79.45 ± 3.21%, respectively. No significant difference was observed between 36 h and 48 h in the DPPH· assay (*p* > 0.05).

The consistent trend between the two independent assays confirmed that enhanced FBRS antioxidant activity was due to bioactive compound accumulation during fermentation. This trend synchronized with the accumulation of anthocyanin aglycones (e.g., cyanidin) and quercetin, released via microbial β-glucosidase-catalyzed glycoside hydrolysis, which constitute the core material basis for FBRS antioxidant activity.

### 3.8. Cell Activity of FBRS

**Figure 6:**
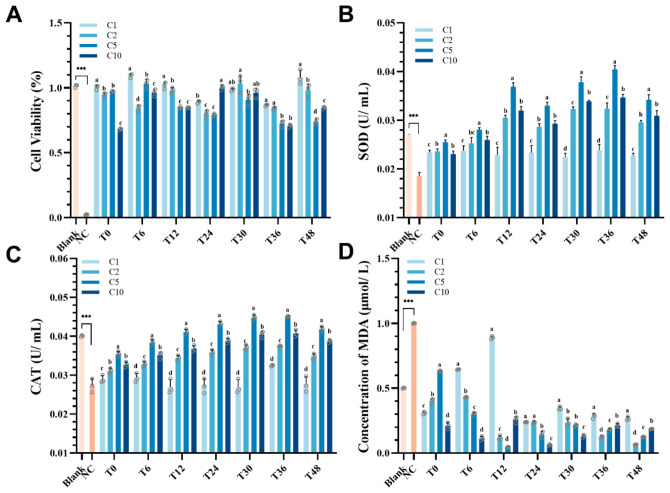
Protective effects of FBRS against H_2_O_2_-induced oxidative stress in Caco-2 cells. (**A**) Cell viability; (**B**) intracellular superoxide dismutase (SOD) activity; (**C**) intracellular catalase (CAT) activity; (**D**) intracellular malondialdehyde (MDA) content. C1/2/5/10, FBRS concentrations of 1%, 2%, 5%, and 10% in DMEM medium; NC, H_2_O_2_-treated negative control group. Data are mean ± SD of triplicate experiments. *** *p* < 0.001 vs. NC group. Different letters indicate significant differences between groups. The NC group is shown in orange to better distinguish between groups. The gray circles in the figure represent the three measured data points from the parallel experiments.

As shown in Figure 6A, cell viability assays revealed that the FBRS extract exerted a dose-dependent protective effect against H_2_O_2_-induced oxidative damage in Caco-2 cells, with the most prominent protective activity observed at 5% concentration (C5) in samples fermented for 30–36 h. The MTT assay results were consistent with the metabolomics findings, which showed simultaneous enrichment of antioxidant compounds in the same fermentation window. This indicated that co-fermentation with *L. bulgaricus 134* and *S. cerevisiae* improved the bioactivity of black rice slurry via biotransformation, and conferred antioxidant activity at the cellular level.

### 3.9. Effects of FBRS on the Contents of MDA, SOD and CAT in the Supernatant of Caco-2 Cells Induced by H_2_O_2_

FBRS pretreatment reversed H_2_O_2_-induced inhibition of SOD activity in a dose-dependent manner (Figure 6B), with the most prominent effects observed in samples fermented for 30–36 h at 5% concentration (C5). This result was consistent with the MTT assay findings (Figure 6A), collectively indicating that FBRS scavenged reactive oxygen species via direct supplementation of antioxidant components, and enhanced cellular oxidative stress resistance by upregulating the endogenous antioxidant enzyme system. CAT activity assays (Figure 6C) revealed that FBRS pretreatment reversed H_2_O_2_-induced loss of CAT activity. The coordinated upregulation of SOD and CAT further confirmed that FBRS supplemented exogenous antioxidant compounds and activated the cellular endogenous antioxidant enzyme system to scavenge superoxide anions and hydrogen peroxide. MDA content measurement (Figure 6D) showed that FBRS pretreatment inhibited H_2_O_2_-induced lipid peroxidation in a dose-dependent manner and reduced MDA production. The effective fermentation window was 30–36 h, with the optimal concentration (C5) consistent with the results of cell viability, SOD and CAT assays.

Collectively, these results indicate that FBRS attenuated H_2_O_2_-induced oxidative injury via supplementing antioxidant compounds and enhancing the endogenous antioxidant defense system, thus maintaining cellular redox homeostasis. This dual antioxidant mechanism was closely related to the peak accumulation of bioactive compounds during the mid-fermentation stage. The regulation of key antioxidant enzymes and inhibition of lipid peroxidation highlight the potential of FBRS as a functional antioxidant ingredient for the intervention of oxidative stress-related disorders.

## 4. Conclusions

The optimal temperature for low-temperature co-fermentation of black rice slurry is 20 °C. At 20 °C, acid produced steadily, while reductive sugars and polyphenols are consumed relatively slowly, which prevents the thermal degradation of anthocyanins.

The maximum extraction of polyphenols and anthocyanins was achieved by fermentation at 20 °C for 36 h. The polyphenols were mainly composed of bound phenols and free anthocyanins, and this composition corresponded to the peak activity of β-glucosidase. As for flavor components, low temperatures have been shown to upregulate metabolic pathways associated with terpenes and esters. This upregulation promoted the extraction of these compounds, resulting in a flavor profile characterized by fruity and sweet notes. The results of both in vitro and in vivo studies indicate the presence of significant antioxidant activity. The fermentation black rice slurry showed the highest ABTS radical scavenging activity at 36 h demonstrated by FBRS. When FBRS was added to DMEM medium at a concentration of 5%, it provided robust protection against H_2_O_2_-induced oxidative stress in Caco-2 cells, maintaining high cellular viability and levels of superoxide dismutase and catalase while significantly reducing malondialdehyde production. This study establishes a novel low-temperature co-fermentation strategy using *S. cerevisiae* and *L. bulgaricus 134* that simultaneously optimizes the flavor profile and functional properties of black rice slurry. By elucidating the underlying metabolic mechanisms driving polyphenol bioconversion and aroma compound generation, this work provides a scientific foundation for developing next-generation fermented grain products with enhanced nutritional value and sensory acceptability.

## Figures and Tables

**Table 1:** Time-dependent changes in the content of differential metabolites of anthocyanins. Different letters of individual metabolites of anthocyanins represent significant differences (*p* < 0.05).

Time/h	Cyanidin/ng	Peonidin/ng	Rutin/ng	Quercetin/ng
0	52,397.34 ± 601.68 ^c^	174.94 ± 4.18 ^e^	185.99 ± 6.28 ^de^	42.54 ± 0.12 ^e^
6	42,643.65 ± 1525.69 ^e^	227.95 ± 7.24 ^d^	204.29 ± 4.73 ^bc^	27.84 ± 0.39 ^f^
12	19,162.08 ± 447.60 ^g^	365.83 ± 9.72 ^b^	223.37 ± 4.20 ^a^	31.54 ± 0.27 ^g^
24	29,265.52 ± 611.70 ^f^	216.06 ± 2.06 ^d^	179.55 ± 3.66 ^e^	62.99 ± 0.80 ^d^
30	57,817.66 ± 1368.88 ^b^	464.69 ± 5.78 ^a^	224.57 ± 2.87 ^a^	84.10 ± 1.58 ^c^
36	62,145.64 ± 1235.54 ^a^	324.60 ± 1.88 ^c^	195.12 ± 0.55 ^cd^	107.94 ± 1.59 ^a^
48	46,583.92 ± 164.15 ^d^	145.10 ± 1.88 ^f^	209.45 ± 6.20 ^b^	100.00 ± 2.51 ^b^

**Table 2:** Time-dependent changes in the content of differential metabolites of polyphenols. Different letters of individual metabolites of polyphenols represent significant differences (*p* < 0.05).

Time/h	Vanillic Acid/ng	Epicatechin/ng	3,4-Dihydroxybenzoic Acid/ng	p-Hydroxycinnamic Acid/ng
0	2293.27 ± 3.24 ^e^	1.39 ± 0.04 ^d^	18,924.53 ± 91.29 ^a^	622.78 ± 0.73 ^bc^
6	2459.08 ± 40.94 ^bc^	0.44 ± 0.02 ^f^	18,432.04 ± 90.42 ^b^	638.95 ± 8.64 ^b^
12	2615.96 ± 39.11 ^a^	1.20 ± 0.03 ^e^	7735.06 ± 63.09 ^c^	683.58 ± 4.7 ^a^
24	2472.28 ± 43.53 ^b^	1.21 ± 0.02 ^e^	5852.63 ± 23.33 ^f^	634.78 ± 15.02 ^b^
30	2556.05 ± 5.69 ^a^	1.74 ± 0.01 ^c^	6820.57 ± 104.43 ^d^	603.41 ± 7.8 ^c^
36	2350.83 ± 30.07 ^de^	3.60 ± 0.12 ^b^	6244.98 ± 18.41 ^e^	562.29 ± 4.35 ^d^
48	2380.17 ± 36.85 ^cd^	7.84 ± 0.12 ^a^	7083.13 ± 103.04 ^d^	442.03 ± 6.62 ^e^

## Data Availability

The original contributions presented in this study are included in the article/Supplementary Materials. Further inquiries can be directed to the corresponding author.

## Supplementary Materials

The following supporting information can be downloaded at: https://www.mdpi.com/article/10.3390/foods15112036/s1, Figure S1: Flavoromics OPLS-DA scatter plot;.Figure S2: Validation of the flavoromics OPLS-DA model; Figure S3: RSD plot for anthocyanin metabolomics data following QC validation; Figure S4: RSD plot for polyphenol metabolomics data following QC validation; Table S1: LCMS results for anthocyanin content; Table S2: LCMS results for polyphenol content.

## Abbreviations

The following abbreviations are used in this manuscript:

DMEM	Dulbecco’s modified eagle medium
FBRS	Fermented black rice slurry
PCA	Principal Component Analysis
OPLS-DA	Orthogonal Partial Least Squares Discriminant Analysis
HCA	Hierarchical Cluster Analysis
MTT	Methylthiazolyldiphenyl-tetrazolium bromide
SOD	Superoxide Dismutase
CAT	Hydrogen Peroxide Enzyme
MDA	Malondialdehyde
KEGG	Kyoto Encyclopedia of Genes and Genomes
