# Supplementary material for: Low-Temperature Co-Fermentation of Lactobacillus bulgaricus 134 and Saccharomyces cerevisiae: Effects on Polyphenols Composition, Flavor Compounds and Antioxidant Activity of Black Rice Slurry
Source: Foods. 2026 Jun 5;15(11):2036. doi: 10.3390/foods15112036 (PMC13256667; doi:10.3390/foods15112036)
Supplement: Supplementary file 1 [file foods-15-02036-s001.zip › foods-4285650-supplementary.pdf]

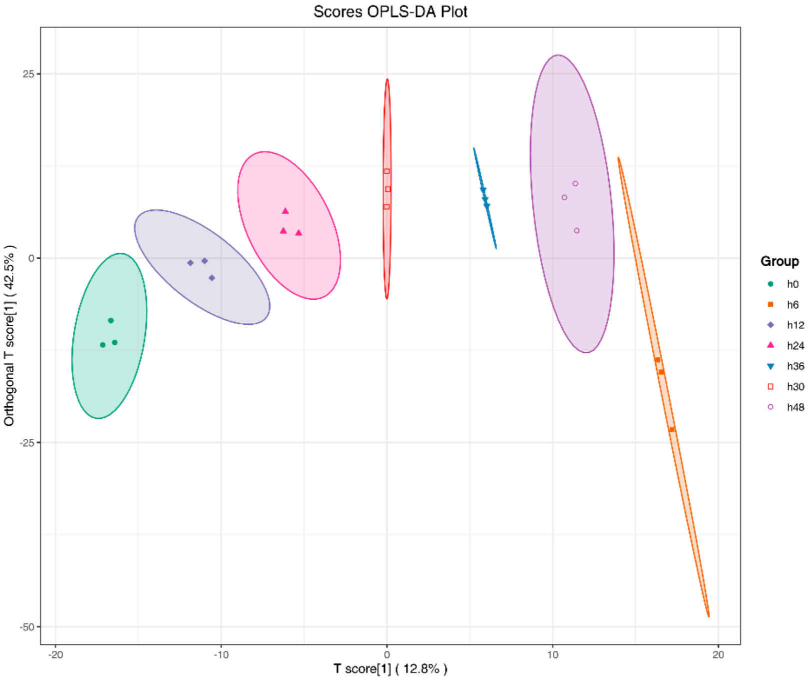

Fig. S1 Flavoromics OPLS-DA scatter plot

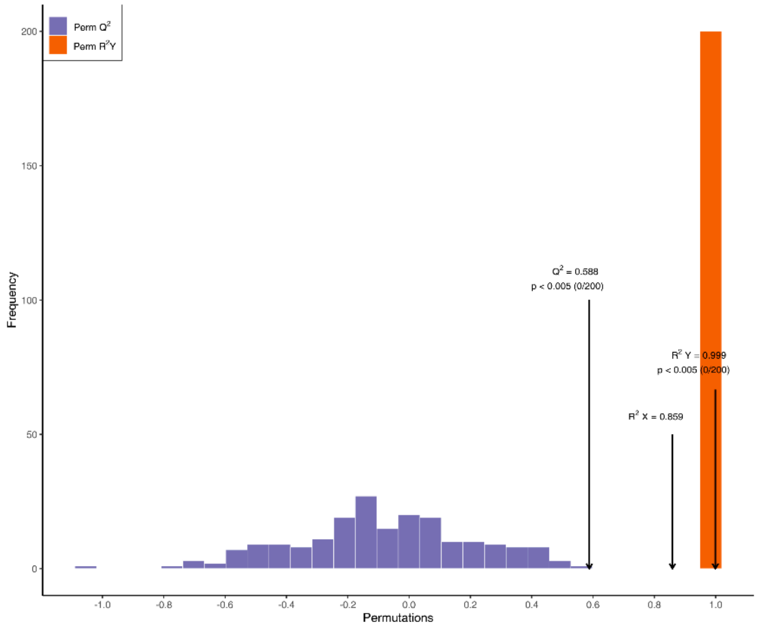

Fig. S2 Validation of the flavoromics OPLS-DA model

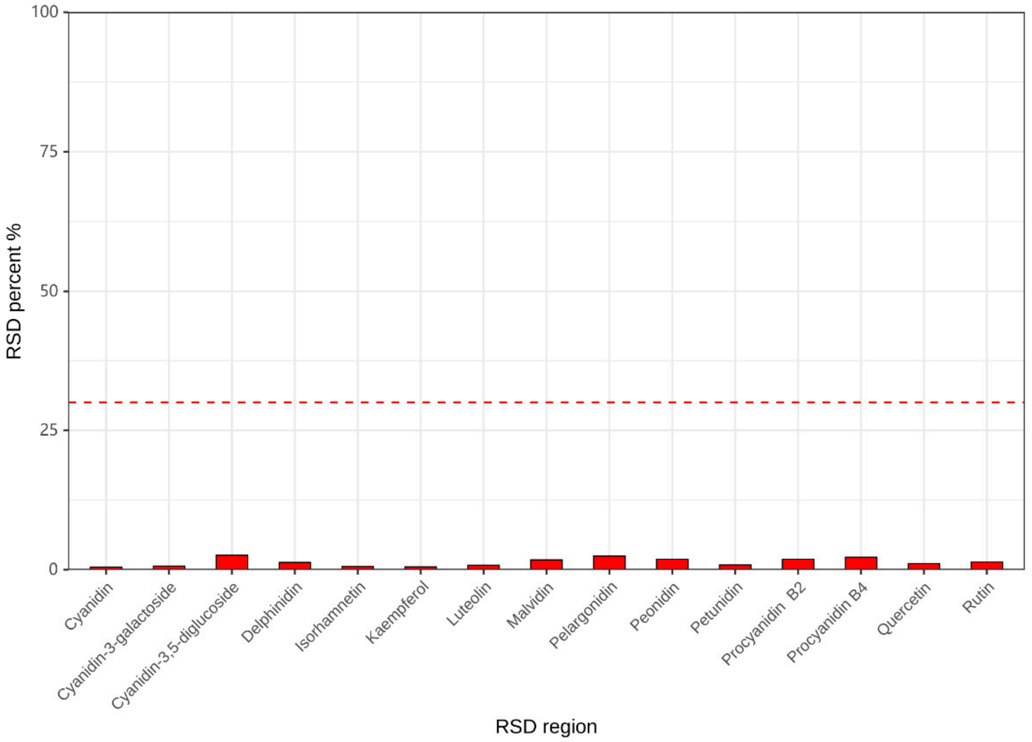

Fig. S3 RSD plot for anthocyanin metabolomics data following QC validation

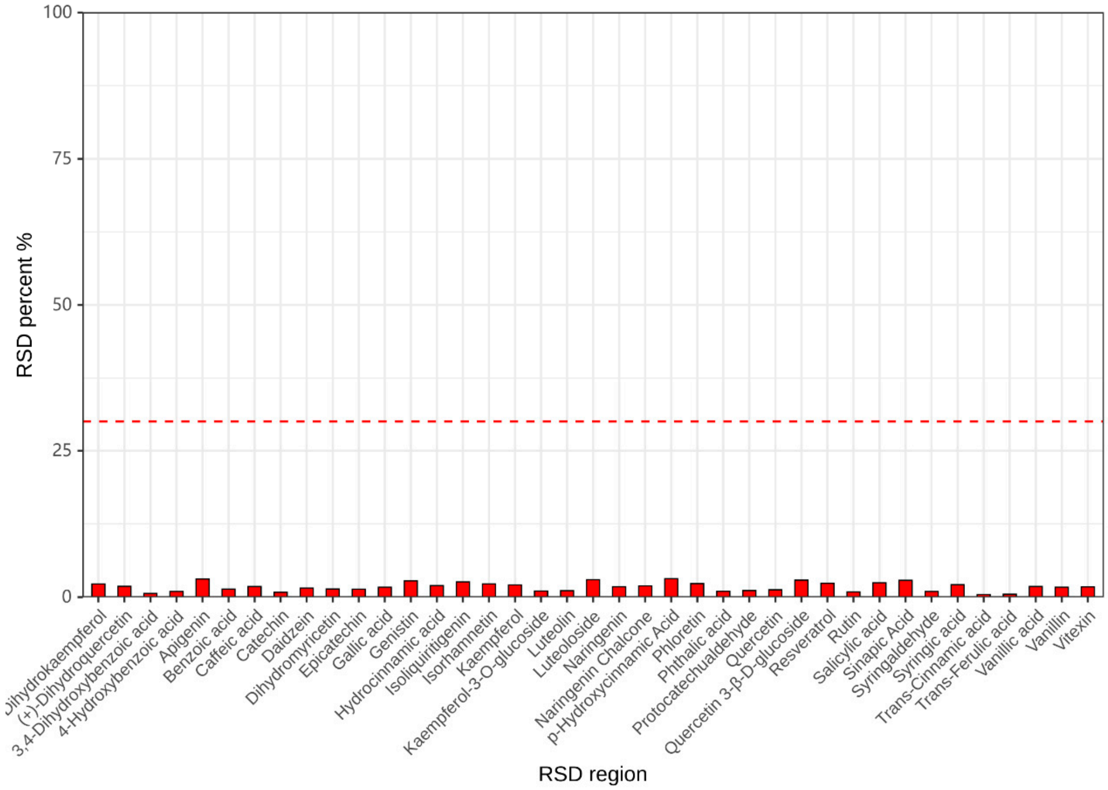

Fig. S4 RSD plot for polyphenol metabolomics data following QC validation

Table S1. LCMS results for anthocyanin content

Time/h	Cyanidin-3,5-diglucoside	Cyanidin-3-galactoside	Delphinidin	Cyanidin	Procyanidin B4
0	0±0	0±0	0±0	52397.3367±601.68	0±0
6	0±0	0±0	0±0	42643.6493±1525.69	0±0
12	0±0	0±0	0±0	19162.0827±447.60	0±0
24	0±0	0±0	0±0	29265.5213±611.70	0±0
30	0±0	0±0	0±0	57817.6753±1368.88	0±0
35	0±0	0±0	0±0	62145.6387±1235.54	0±0
48	0±0	0±0	0±0	46583.9200±164.15	0±0

Time/h	Procyanidin B2	Petunidin	Pelargonidin	Peonidin	Malvidin
0	0±0	26.2940±0.37	7.8240±0.15	174.9433±4.18	15.4347±0.42
6	0±0	25.8293±0.40	8.3573±0.29	227.9527±7.24	0.0000±0.00
12	0±0	25.0593±0.61	2.5433±0.05	145.0993±1.88	9.4100±0.09
24	0±0	23.5680±0.36	4.9533±0.03	216.0587±2.06	4.5000±0.05
30	0±0	29.3253±0.31	8.8867±0.08	365.8293±9.72	0.0000±0.00
35	0±0	25.5487±0.36	7.5807±0.05	464.6907±5.78	0.0000±0.00
48	0±0	30.9267±0.18	5.5807±0.12	324.5987±1.88	5.5567±0.08

Time/h	Rutin	Luteolin	Quercetin	Isorhamnetin	Kaempferol
0	185.9887±6.28	5.1013±0.09	42.5360±0.12	5.2093±0.06	2.2420±0.08
6	204.2853±4.73	5.4027±0.06	27.8427±0.39	4.6887±0.12	3.6333±0.12
12	209.4547±6.20	5.6533±0.12	100.0027±2.51	12.8913±0.67	4.1033±0.04
24	179.5540±3.66	6.6233±0.34	62.9873±0.80	9.7913±0.23	5.9773±0.08
30	223.3673±4.20	4.7613±0.17	31.5447±0.27	4.8680±0.04	3.3173±0.06
35	224.5733±2.87	6.7033±0.24	84.1087±1.58	11.3967±0.25	3.7280±0.12
48	195.1213±0.55	7.4727±0.05	107.9367±1.59	15.1200±0.19	2.3200±0.05

Table S2. LCMS results for polyphenol content

Time(h)	Vanillic Acid	4-Hydroxybenzoic Acid	Benzoic Acid	Phthalic Acid
0	2293.2733±3.24	240.5027±1.79	226.5793±1.82	131.2447±0.54
6	2459.0833±40.94	222.49±5.68	226.2967±4.82	61.2373±2.70
12	2615.9593±39.11	226.5347±7.55	213.6787±1.21	62.126±2.26
24	2472.282±43.53	219.5787±2.46	173.5573±4.95	56.7053±1.55
30	2556.0467±5.69	225.5693±4.35	179.1533±4.01	58.088±1.91
36	2350.8293±30.07	202.64±4.65	175.816±2.14	44.088±0.88
48	2380.1687±36.85	183.212±6.19	162.3073±4.47	42.2693±0.97

Time(h)	Epicatechin	Naringenin	Dihydromyricetin	(+)-Dihydrokaempferol
0	1.3907±0.04	8.2093±0.08	41.5273±1.21	3.0173±0.05
6	0.444±0.02	13.22±0.34	48.9807±2.18	3.9447±0.06
12	1.2007±0.03	15.636±0.13	50.0267±1.76	4.5607±0.07
24	1.2133±0.02	14.3727±0.29	45.932±0.97	3.758±0.1
30	1.7427±0.01	21.8067±0.45	55.984±0.87	4.1547±0.21
36	3.6±0.12	16.844±0.12	43.4913±1.15	4.1253±0.14
48	7.844±0.12	17.868±0.07	47.4173±0.88	4.0893±0.12

Time(h)	Hydrocinnamic Acid	3,4-Dihydroxybenzoic Acid	Isorhamnetin	Genistin	Daidzein	Resveratrol
0	4.2767±0.11	18924.5293±91.29	5.4053±0.11	21.196±1.88	30.872±0.8	2.7533±0.17
6	4.7773±0.12	18432.0433±90.42	8.6133±0.06	23.55±1.78	37.6007±1.15	4.8153±0.19
12	3.7047±0.07	7735.0607±63.09	12.7973±0.28	23.2747±1.6	43.5867±1.4	5.5387±0.16
24	3.9927±0.14	5852.6287±23.33	8.384±0.27	21.0307±1.39	39.5687±0.59	5.0873±0.06
30	4.3733±0.07	6820.5733±104.43	14.12±0.22	19.8793±0.76	47.006±1.52	8.378±0.16
36	3.692±0.19	6244.9847±18.41	9.2313±0.16	13.514±0.3	42.6593±0.47	5.4107±0.13

48	3.762±0.03	7083.1253±103.04	9.8213±0.11	0±0	41.5067±0.16	6.726±0.14

Time(h)	Ferulic Acid	Caffeic Acid	p-Hydroxycinnamic Acid	Sinapic Acid	Apigenin	Naringenin
0	705.19±1.86	52.8253±0.45	622.7807±0.73	124.842±0.61	18.2733±0.23	8.2093±0.08
6	561.804±11.3	27.276±1.32	638.9507±8.64	127.514±0.74	28.168±0.63	13.22±0.34
12	581.006±3.08	33.584±7.68	683.5767±4.7	117.614±2.63	31.9393±0.3	15.636±0.13
24	544.668±6.85	31.9593±2.84	634.78±15.02	128.2253±2.84	26.8847±0.37	14.3727±0.29
30	534.6193±2.82	41.366±3.19	603.4113±7.8	131.236±1.41	38.0107±0.63	21.8067±0.45
36	495.5827±10.36	37.6967±2.76	562.2873±4.35	124.1227±2.59	28.9467±0.18	16.844±0.12
48	423.896±14.37	38.3753±1.18	442.028±6.62	114.6827±1.9*	28.5833±0.74	17.868±0.07
